# Metagenomic next-generation sequencing provides prognostic warning by identifying mixed infections in nocardiosis

**DOI:** 10.3389/fcimb.2022.894678

**Published:** 2022-08-31

**Authors:** Mengfan Jiao, Xiaoxu Ma, Yaoguang Li, Huifen Wang, Ying Liu, Wenhu Guo, Jun Lv

**Affiliations:** ^1^ Department of Infectious Diseases, The First Affiliated Hospital of Zhengzhou University, Zhengzhou, China; ^2^ Gene Hospital of Henan Province, The First Affiliated Hospital of Zhengzhou University, Zhengzhou, China; ^3^ Department of Respiration, The First Affiliated Hospital of Zhengzhou University, Zhengzhou, China; ^4^ R&D Center, Agene (Fuzhou) Medical Laboratory Co. Ltd., Fuzhou, China

**Keywords:** *Nocardia*, metagenomic next-generation sequencing (mNGS), precise treatment, mixed infection, infectious diseases

## Abstract

*Nocardia* is an opportunistic pathogen that mainly involves immunosuppressed patients and causes a high mortality rate. As an emerging approach to detect infectious pathogens, metagenomic next-generation sequencing (mNGS) was reported in the detection of *Nocardia*. However, there is no evidence demonstrating the effect of mNGS on the prognosis of *Nocardia* infection. In this retrospective study, we included 18 nocardiosis patients. *Nocardia* species were detected by mNGS from their clinical samples. All the patients were diagnosed with nocardiosis by clinical experts through a comprehensive evaluation. Of these 18 patients, fever is the most frequent initial symptom. Compared to traditional culture methods, mNGS provides a faster turnaround time (TAT) and higher sensitivity. Pulmonary nocardiosis was the most common clinical presentation in the study. mNGS detected 13 types of *Nocardia* species, of which *Nocardia abscessus* and *Nocardia cyriacigeorgica* were the most common species. The study’s most noteworthy discovery is that mNGS outperforms culture at detecting mixed infections (more than one pathogen detected in one clinical specimen, including bacteria, fungi, and excluding virus), and number of infectious species was an independent risk factor for nocardiosis patients’ prognostics after adjusting age, ICU days, gender and underlying diseases (adjusted HR = 1.47, 95% CI: 1.09-1.98, p = 0.011). As a result, we believe that by detecting mixed infections (more than one pathogenic species), mNGS can provide a clinical risk warning for the prognosis of nocardiosis.

## Introduction

Nocardiosis is a rare infection caused by the genus Nocardia, a Gram-positive, weakly acid-fast, and aerobic bacteria, which belongs to the order Actinomycetales (McNeil and Brown, 1994a). As an opportunistic pathogen, Nocardia mainly causes infections in immunosuppressed patients (Beaman et al., 1976; Rathish B Zito, 2020). Nocardiosis is a relatively rare infectious disease. A study which was the largest research reported 441 Nocardia strains in 21 provinces from 2009 to 2021 in China (Wang et al., 2022). In a tuberculosis hospital in Amritsar, the prevalence of pulmonary nocardiosis was 1.4% (Singh et al., 2000). The manifestations of Nocardiosis are in a wide range and are mainly decided by the infection sites, which are serious and atypical clinical (Wilson, 2012). Early diagnosis and treatment of pulmonary nocardiosis promote favorable clinical prognoses (Buckley et al., 1995). The gold standard for nocardiosis laboratory diagnosis is bacterial culture, which usually takes 3-5 days and the sensitivity may be decreased by prior antibiotics usage (Rouzaud et al., 2018a; Rathish, 2020). A former study reported 16S rRNA PCR as a rapid assay for detecting Nocardia, but the approach requires clinicians’ pre-consideration (Couble et al., 2005).

The prognosis of Nocardia is highly dependent on early pathogen identification and drug therapy. Sulfonamides are the first-line treatment of nocardiosis after the first use in the 1940s (Welsh et al., 2013). Before the discovery of the treatment, the mortality rate of pulmonary nocardiosis was close to 100% and was much higher than that of other bacterial infections (Brown-Elliott et al., 2006). In fact, due to the relative rarity of Nocardia infection and the lack of effective detection methods, the best treatment period may have been missed, resulting in a poor clinical outcome (Yagi et al., 2014).

Metagenomic next-generation sequencing (mNGS) is a new approach by sequencing DNA or RNA fragments to identify theoretically all infectious pathogens in clinical specimens (Simner et al., 2018; Gu et al., 2019). mNGS shows high sensitivity and specificity and can be less influenced by former antibiotics usage, which is beneficial for complex infection and fever of unknown origin (FUO) (Goldberg et al., 2015; Miao et al., 2018; Fu et al., 2022). Furthermore, mNGS is more efficient and outperforms traditional microbiological culture when detecting multiple infections (Chen et al., 2021).

A study in 2019 reported the sensitivity of mNGS for Nocardia detection was superior to that of conventional culture methods (Weng et al., 2020). However, there was no study showing the prognostic effects of mNGS in nocardiosis.

In this retrospective study, we included 18 cases who were detected Nocardia species by mNGS and diagnosed as nocardiosis by clinical specialists through composite reference standards. We discovered that mNGS exhibited higher sensitivity and faster turnaround time (TAT) than traditional laboratory methods. We significantly noticed that the number of infectious species was an independent risk factor for nocardiosis patients. In addition, mNGS outperformed traditional microbiological culture in the detection of mixed infections. By detecting mixed infections, mNGS can provide early warning for nocardiosis patients.

## Materials and methods

### Patient recruitment

Between May 2020 and July 2021, 18 participants were enrolled in this retrospective study at the First Affiliated Hospital of Zhengzhou University from May 2020 and July 2021. We recruited patients who were detected with Nocardia species from their clinical specimens by metagenomic next-generation sequencing (mNGS) and diagnosed with Nocardia infection by clinical specialists through composite reference standards. The composite reference standards were based on clinical manifestations, laboratory tests, microbiological tests, and radiography. Patients who were identified Nocardia species by mNGS but were not clinically diagnosed, together with minor patients were excluded from the study. Totally there were 18 patients enrolled in this study. Every specimen was performed both by mNGS and microbiological culture. Patients’ information, including demographic characteristics, clinical history, laboratory tests, mNGS, and microbiological culture information were collected in the hospital information systems.

### Specimen collection

Five types of samples were collected from the patients. There were 10 bronchoalveolar lavage fluid (BALF) samples, 4 blood samples, 2 cerebrospinal fluid (CSF) samples, 1 lung tissue sample, and 1 abscess sample. The lung tissue sample size was more than 3X3X3mm3, and the samples were transported in a 5ml cryopreservation tube on dry ice. More than 5ml of bronchoalveolar lavage fluid (BALF) and abscess were collected and stored in a 50ml cryopreserved tube. Cerebrospinal fluid (CSF) was collected at least 1ml and stored in a 5ml cryopreserved tube. BALF, abscess, and CSF were all transported with dry ice. Blood samples were collected more than 4ml in ethylene diamine tetraacetic acid blood collection tubes and were transported with ice packs.

In this study, we gathered mNGS and microbiological culture information, as well as some time nodes. There are some time nodes defined in our study: specimen time, from the first hospital day to sample collection day; turnaround time (TAT), from specimens submitted for mNGS or microbiological culture to receive the reports; results time, from the first hospital day to receive the mNGS or microbiological culture reports.

### DNA/RNA extraction, library construction and sequencing

DNA was extracted using the QIAamp® UCP Pathogen Kit (Qiagen, Germany) according to the manufacturer’s protocol. Cell-free DNA (cfDNA) from plasma samples was extracted by using the TIANamp Micro DNA DP316 Kit (Tiangen Biotech, Beijing, China) following the manufacturer’s recommendations.

The extracted DNA samples were used to construct DNA libraries by using the TruePrep DNA Library Prep Kit V2 for Illumina® (Vazyme, Nanjing, China). cfDNA samples were used to construct DNA libraries by using the VAHTS Universal DNA Library Prep Kit V3 for Illumina® (Vazyme, Nanjing, China). All libraries were prepared following the manufacturer’s manuals. The Agilent 2100 Bioanalyzer (Agilent Technologies, Santa Clara, USA) was used for library quality control. All libraries were pooled with other libraries by using different index sequences and sequenced on an Illumina NextSeq 550Dx platform with the single-end 75bp sequencing option. For each run, no-template control (NTC) samples (Nuclease-free H2O) were also pooled to monitor reagent and laboratory background.

### Bioinformatics analysis

Fastq-format data were obtained for each sample by using bcl2fastq software (v2.20.0.422, parameters used: –barcode-mismatches 0 –minimum-trimmed-read-length 50). Adapt sequences and low-quality reads were filtered out using cutadapt v2.10 (-q 25, 25 -m 50). The remaining high-quality reads were first mapped to the human genome (hg38, https://hgdownload.soe.ucsc.edu/downloads.html#human) using bwa-mem 2 v2.1 with default parameters, all unmapped reads were then aligned to the NCBI nt database (https://ftp.ncbi.nlm.nih.gov/genomes/) by using BLAST v2.9.0+ (-task megablast -num_alignments 10 -max_hsps 1 -evalue 1e-10). Alignments were required to be full-length with an identity of at least 95%. A customized Python script was used to identify species-specific alignments. Only the alignments that fulfill the above-mentioned criteria were used for further pathogen identification. Besides, the NTC samples were used to identify reagent and laboratory contaminants. Microorganisms were reported if the following criteria were met: (1) the microbe had at least 3 non-redundant, mapped reads per 10 million raw sequence reads, and (2) the microbe was known to be potentially pathogenic in the given clinical context of each patient.

### Statistical analysis

Statistical analyses were all performed on R version 3.6.1. Continuous variables, shown as mean (standard deviation), were compared using Wilcoxon rank-sum test between two groups. Categorical variables, shown as counts and percentages [n (%)], were compared using Fisher’s exact test. Statistical significance was considered as a two-sided p-value <0.05. Patients’ hospital costs and antibiotic costs were calculated by “scale” package of R based on the actual cost. Cox proportional hazards model was applied for discovering the independent risk factors for nocardiosis. The multivariate model was included variables which was significant in univariate analysis and variables which were commonly important in patients’ outcomes. Survival curves were adjusted for age, ICU days, gender and underlying diseases.

## Results

### Patients’ characteristics

All the nocardiosis patients’ characteristics were summarized in **Table 1**. Among the 18 patients, there were 14 men and 4 women. Patients’ age ranged from 28 to 76 with an average age of 56.78. Ten of them were over 60 years old. Ten patients were admitted to the intensive care unit (ICU) and their mean ICU length of 10.2 days. Six patients required ventilator ventilation for respiratory support. The antibiotic percentage was calculated as the ratio of antibiotic expenditure to overall hospitalization expenditure. Antibiotics accounted for 16% of overall hospital costs on average. Fever was the most prevalent symptom in this study (72.22%, 13/18), followed by cough (27.78%, 5/13) and chest tightness (27.78%, 5/13). Fifteen of the patients had underlying diseases and 9 of them had more than one kind illnesses. Hypertension, diabetes, and pulmonary disease were all prevalent and each of them accounted for 27.78% (5/13). There were 2 hepatitis C patients and 1 malignant tumor patient. Disseminated nocardiosis was considered as a simultaneous infection of at least two non-contiguous organs, or Nocardia bacteremia. For different nocardiosis sites, 11 patients suffered pulmonary nocardiosis, followed by disseminated nocardiosis in 5 patients (2 patients suffered Nocardia bacteremia and pulmonary nocardiosis; 2 patients occurred Nocardia bacteremia; the other patient developed cutaneous and pulmonary nocardiosis). Central nervous system (CNS) infection was found in 2 patients (**Table S1**).

**Table 1:** Patients’ characteristics.

Characteristics	Total (n=18)
**Age, mean ± sd**	56.78 ± 14.19
**Gender, no. (%)**	
Male	14 (77.78)
Female	4 (22.22)
**ICU, no. (%)**	10 (55.56)
ICU days	10.20 ± 8.32
**Ventilator, no. (%)**	6 (33.33)
Ventilator hours	180.67 ± 77.18
**Hospital days, no. (%)**	
<10 days	5 (27.78)
10-30days	6 (33.33)
>30days	7 (38.89)
**Main symptoms, no. (%)**	
Fever	13 (72.22)
Cough	5 (27.78)
Chest tightness	5 (27.78)
Chest pain	3 (16.67)
Dyspnea	2 (11.11)
Local swelling	1 (5.56)
**Underlying diseases, no. (%)**	
Hypertension	5 (27.78)
Diabetes	5 (27.78)
Pulmonary disease	5 (27.78)
CKD	4 (22.22)
Coronary heart diseases	3 (16.67)
Hepatitis C	2 (11.11)
Malignant tumor	1 (5.56)
**Nocardia infection sites, no. (%)**	
Pulmonary	11 (61.11)
Disseminated	5 (27.78)
CNS	2 (11.11)

ICU, intensive care unit; CKD, chronic kidney disease; CNS, central nervous system.

### The comparison between mNGS and microbiological culture results

Every patient’s specimen was performed with both microbiological culture and mNGS. In this study, 18 specimens were classified into 5 different types (10 bronchoalveolar lavage fluid (BALF) specimens, 4 blood samples, 2 cerebrospinal fluid (CSF) samples, 1 lung tissue sample, and 1 abscess sample) (**Table 2**). The average specimen time was 3.33 days. Ten patients’ mNGS TAT was only 1 day, compared to 2 days of culture TAT in 16 samples. The average mNGS TAT was significantly shorter than that of culture (average time, 1.33 and 2.78 days, respectively, P<0.001).

**Table 2:** mNGS and microbiological culture information.

Characteristics	Total (n=18)	P value
**Specimen types, no. (%)**	** **	
BALF	10 (55.56)	
Blood	4 (22.22)	
CSF	2 (11.11)	
Lung tissue	1 (5.56)	
Abscess	1 (5.56)	
**Specimen time, mean ± sd**	3.33 ± 4.21	
**mNGS turnaround time day, mean ± sd**	1.33 ± 0.77	0.001
**Culture turnaround time day, mean ± sd**	2.78 ± 1.35	
**mNGS result time day, mean ± sd**	4.67 ± 4.12	0.372
**Culture result time day, mean ± sd**	6.06 ± 5.05	

CSF, Cerebrospinal Fluid.

Only 5 specimens were culture-positive among the 18 mNGS-positive patients, and only 3 specimens were cultured with Nocardia (**Table S2**). Species Candida tropicalis and Klebsiella pneumoniae were found in the other two culture-positive specimens, which were also identified by mNGS. Here, we defined the mixed infections in this study as one patient infected with more than one pathogenic species (including bacteria, and fungal but excluding virus). In this study, microbiological culture only detected single species, the results of which were all verified by mNGS. mNGS outperformed culture at detecting mixed infections.

### Characteristics of mNGS results

**Figure 1:**
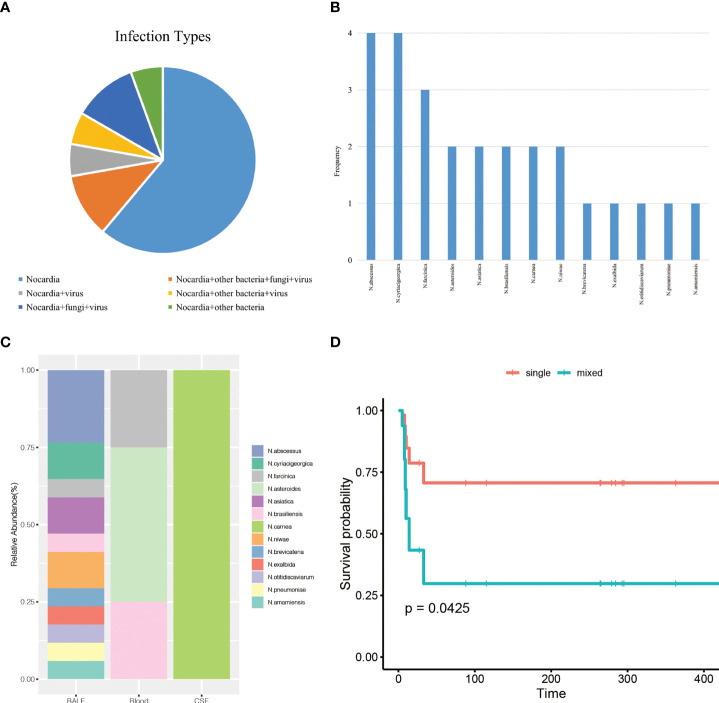
**(A)**, infection types of the patients. **(B)**, the frequency of the 13 *Nocardia* species detected by mNGS. **(C)**, the distribution of *Nocardia* species in BALF, blood, and CSF samples. **(D)**, overall survival analysis for nocardiosis patients according to infection types.

Among the 18 patients, mNGS detected 13 Nocardia species, 8 other bacteria, 4 fungi, and 6 viruses (**Table S2**). The main infection type in the patients was simple Nocardia infection in 11 patients (**Figure 1A**). Seven patients were mixed infections of Nocardia accompanied by other bacteria, fungi, or viruses. Infections of Nocardia accompanied by other bacteria, fungi, together with viruses were found in two patients. Four patients were mixed infections consisting of Nocardia and fungi, with 3 of them having chronic kidney diseases (**Table S2**). Pneumocystis jirovecii was the most frequent fungus in the mixed infections patients.

In this study, mNGS detected 13 different Nocardia species (**Figure 1B**). Four patients were detected with only one Nocardia species, with at least 2 Nocardia species isolated in the remaining 14 patients. Nocardia abscessus and Nocardia cyriacigeorgica were more frequently occurring in 4 patients, followed by Nocardia farcinica (3 patients) and Nocardia asteroids (2 patients).

The distribution of Nocardia species in BALF, blood, and CSF was shown in **Figure 1C**. Apparently, Nocardia species diversity was increased in BALF samples compared with blood and CSF samples. In our study, it was also noticed that some Nocardia species only existed in one single type specimen. N.abscessus, for example, was only detected in BALF. N.asteroides was only observed in blood and Nocardia carnea was only detected in CSF. However, this effect may occur by chance because of the limited sample numbers.

### The use of antimicrobial drugs

All the patient’s antimicrobial drugs were collected (**Table S3**). According to the patient’s antimicrobial drug usage and the classification of antibiotics, we grouped the patient’s drugs into 10 primary categories, including sulfonamides, oxazolidinones, carbapenems, penicillin, β-lactamase inhibitors, antifungal drugs, cephalosporin, tetracyclines, fluoroquinolones, and others. Totally 18 antimicrobial drugs were administrated. During their hospital stay, each patient received an average of 4-5 types of antibiotic treatments. Among the antimicrobial drugs, trimethoprim-sulfamethoxazole (TMP-SMX), biapenem, voriconazole, linezolid, and imipenem were most commonly used.

### The comparison of characteristics between survivors and non-survivors and survival analysis

The data cutoff date for the survival analysis was July 26, 2021. We obtained all the patients’ follow-up information. Among the 18 patients, there were 11 survivors and 7 non-survivors. The baseline information of the two groups were shown in **Table 3**. There was no difference in age, gender, underlying diseases, and Nocardia infection sites between the two groups. Non-survivors showed significantly more infectious species numbers than those of survivors (average number, 1.273 and 4, respectively, P=0.006). Non-survivors were more likely admitted into ICU and placed on ventilators as a result of the severe condition.

**Table 3:** The comparison of characteristics between survivors and non-survivors.

Feature	Survivors (n=11)	Non-survivors (n=7)	P value
**Age**	51.818 (15.728)	64.571 (6.503)	0.07
**Gender**			
female	2	2	1
male	9	5	
**Underlying disease**			
no	2	1	1
yes	9	6	
**Underlying disease numbers**	1.818 (0.982)	2 (1.633)	1
**Numbers of infectious species**	1.273 (0.467)	4 (3.464)	0.006
***Nocardia* infection sites**			
pulmonary	6	5	0.793
disseminated	4	1	
CNS	1	1	
**Hospital days**	28.182 (13.659)	20.571 (22.53)	0.123
**ICU or not**			
no	8	0	0.004
yes	3	7	
**ICU days**	14.333 (15.695)	8.429 (3.101)	0.908
**Ventilator or not**			
no	11	1	0
yes	0	6	
**Specimen information**			
mNGS turnaround time day	1.364 (0.924)	1.286 (0.488)	0.755
mNGS result time day	5.818 (4.956)	2.857 (0.9)	0.23
Specimen time	4.455 (5.145)	1.571 (0.535)	0.509
Culture turnaround time day	2.909 (1.446)	2.571 (1.272)	0.737
Culture result time day	7.364 (6.12)	4 (1.291)	0.265
**Costs information**			
Hospital costs	0.573 (1.035)	0.559 (0.284)	0.085
Antibiotic costs	0.679 (0.861)	0.554 (0.629)	0.659
Antibiotics percentage	0.179 (0.074)	0.119 (0.078)	0.211
Daily hospital costs	0.468 (0.512)	1.158 (0.655)	0.035
Daily antibiotic costs	0.683 (0.504)	1.012 (0.6)	0.285
**Symptom to hospital**	36.2 (42.949)	71 (53.432)	0.094
**Symptom to TMP-SMX**	42.1 (42.969)	72.2 (52.926)	0.198
**TMP-SMX**			
no	1	2	0.528
yes	10	5	
**Laboratory tests**			
CRP	133.824 (107.458)	139.841 (93.304)	0.813
PCT	0.552 (0.422)	4.201 (8.193)	0.299
ESR	57.25 (27.366)	80.6 (41.065)	0.413
Leukocyte	13.432 (6.018)	14.169 (6.753)	1

CNS, central nervous system; ICU, intensive care unit, CKD, chronic kidney disease; mNGS, metagenomic next-generation sequencing; TMP-SMX, trimethoprim-sulfamethoxazole; CRP, C-response protein; PCT, procalcitonin.

We initially included age, ICU days, gender, underlying diseases, and the number of infectious species numbers in the univariate regression analysis (**Table 4**). The evidence was significant that more infectious species numbers were associated with shorter survival (HR = 1.34, 95% confidence interval (CI): 1.07-1.69, p = 0.013). Multivariate analysis suggested the number of infectious species was an independent prognostic marker (adjusted HR = 1.47, 95% CI: 1.09-1.98, p = 0.011). Survival curves separated from single infection and mixed infections after adjusting for age, gender, ICU days and underlying diseases, indicating that patients with mixed infections developed significantly poor prognosis (P = 0.0425) (**Figure 1D**).

**Table 4:** Univariate analysis and multivariate analysis of overall survival in nocardiosis patients.

Characteristics	Univariate analyses			Multivariate analyses		
	HR	95% CI	P value	HR	95% CI	P value
**Age**	1.07	0.99-1.16	0.098	1.07	0.96-1.19	0.239
**Gender**	0.45	0.09-2.35	0.343	0.32	0.04-2.78	0.299
**ICU days**	1.03	0.97-1.1	0.329	1.04	0.93-1.16	0.488
**Numbers of infectious species**	1.34	1.07-1.69	0.013	1.47	1.09-1.98	0.011
**Underlying diseases**	1.33	0.16-11.16	0.793	0.42	0.04-5.03	0.496

HR, hazard ratio; CI, confidence interval.

## Discussion

In this study, we included 18 patients who were detected Nocardia by metagenomic next-generation sequencing (mNGS) and clinically diagnosed with nocardiosis. We compared mNGS and microbiological culture results and we analyzed the demographic characteristics, underlying diseases, infection sites, hospital information, and survival risk factors for nocardiosis patients. Consistent with previous studies, mNGS developed a faster turnaround time (TAT) than microbiological culture and outperformed culture in mixed infections detection (Miao et al., 2018; Chen et al., 2021). More importantly, we discovered that number of infectious species was the only independent survival risk factor for nocardiosis patients. Thus, we tentatively put forward that mNGS can provide a clinical warning by detecting definite infectious species numbers and help in the clinical decision and patients’ prognoses.

Culture has been commonly considered the golden standard for Nocardia detection, but its usefulness was hindered due to the strict culture condition and previous antibiotic treatment (Kerr et al., 1992; Brown-Elliott et al., 2006). Nocardia culture may take 2-7 days even for several weeks, resulting in a delay in establishing the diagnosis (Conville and Witebsky, 2015). The average culture TAT in this study was 2.78 ± 1.35 days, significantly longer than the mNGS TAT (1.33 ± 0.77 days). Besides, the low culture-positive detection rate was also noticed: only 5 of the 18 mNGS-positive samples were positive for culture, with 3 Nocardia-positive samples. A study in 2020 illustrated 5 samples yielded culture-positive among the 14 mNGS-positive samples, and also exhibited a faster mNGS TAT than that of culture, consisting with our findings (Weng et al., 2020). However, there was no prognostic analysis in this study. To the best of our knowledge, we performed the first research to explore the usage of mNGS in prognoses of nocardiosis patients.

When Nocardia species were isolated in respiratory tract samples, the colonization should be taken into consideration. The colonization of Nocardia was defined as a lack of clinical evidence (Margalit et al., 2020). Nocardiosis was defined as a clinically evident infection related to the isolation of the Nocardia species (Margalit et al., 2020). In our study, we recruited patients who were detected with Nocardia species from their clinical specimens by metagenomic next-generation sequencing (mNGS). It was important that all the patients were clinically diagnosed with nocardiosis by clinical specialists through composite reference standards. Therefore, we ruled out the patients with the colonization of Nocardia.

The prognosis of nocardiosis is determined by many factors as previously reported. The early diagnosis and appropriate treatment are key factors related to favorable outcomes (Lerner et al., 1967). Nocardia species usually show susceptibility to these antibiotics: sulfonamides, oxazolidinones, aminoglycosides,β-lactams, quinolones, macrolides, and tetracyclines (Welsh et al., 2013). Before the discovery of the treatment, the mortality rate of pulmonary nocardiosis was reported close to 100%, which was much higher than that of other bacterial infections (Brown-Elliott et al., 2006). Among the above various antibiotics, sulfonamides and linezolid are both frequently used. Sulfonamides have been first discovered as the treatment for nocardiosis in the 1940s and have been administrated as the first-line treatment (Edward P. Benbow et al., 1944). Trimethoprim-sulfamethoxazole (TMP-SMX) is the combination of trimethoprim and sulfamethoxazole and becomes an effective treatment (Wilson, 2012). Linezolid can conduct a good effect in clinical practice, especially in disseminated and central nervous system (CNS) nocardiosis (Moylett et al., 2003). Drug resistance also exists in Nocardia (Welsh et al., 2013). In our study, 15 patients were treated with TMP-SMX and 7 patients were treated with linezolid. Among them, 6 patients received both TMP-SMX and linezolid. Combination therapy of at least two antibiotics is recommended in disseminated nocardiosis (McNeil and Brown, 1994b; Ambrosioni et al., 2010). Compared with immunocompetent patients, immunosuppressed nocardiosis patients may associate a the more severe condition and poorer outcomes (Steinbrink et al., 2018). As a result, the recommended treatment duration for immunosuppressed nocardiosis individuals should last at least 12 months, and treatment for immunocompetent individuals is suggested to be 6-12 months (Wilson, 2012).

Until 2017, 92 Nocardia species have been recognized and 54 of them have been proved to be associated with clinical diseases (Conville et al., 2017). N. asteroides was reported as the main pathogenic taxonomy (Conville et al., 2017). A study investigated clinical features of Nocardia in 7 cities in China between 2009-2017 and reported the majority species as N. farcinica, followed by N. cyriacigeorgica (Huang et al., 2019). In our study, mNGS detected N. abscessus and N. cyriacigeorgica the most, followed by N. farcinica. Whether every clinical detection of Nocardia requires the species level remains unknown. However, it is worth noting that different Nocardia species may develop various phenotypic characteristics and drug sensitivities (Brown-Elliott et al., 2006; Derungs et al., 2021). Traditional methods usually are unable to identify Nocardia species. It is also worth noting the molecular techniques 16S rRNA–based polymerase chain reaction (PCR), with high specificity and sensitivity, can also be used to identify Nocardia (Couble et al., 2005; Rouzaud et al., 2018b). However, 16S rRNA PCR only detects one pathogen at a time. In addition, all samples submitted for 16S rRNA PCR require clinicians to make a pre-consideration for the pathogen, which is unfavorable for infections with atypical clinical manifestations and laboratory examination (Weng et al., 2020). Nevertheless, as a new approach with advantages in detecting infectious pathogens, mNGS can identify Nocardia to the species level, assisting in the typical treatment.

For the comparison of characteristics between survivors and non-survivors, no significant difference was found in age and underlying diseases between non-survivors and survivors. In addition, Nocardia infection sites of non-survivors did not differ from the survivors. A significant difference in the number of infectious species was noticed between survivors and non-survivors. Univariate and multivariate regression analysis discovered that number of infectious species was an independent prognostic risk factor. More infectious species were associated with poor prognosis in nocardiosis patients. Similar results were reported in hematological malignancies. Polymicrobial pulmonary infection is associated with high mortality nearly 50% (Hardak et al., 2016). To our knowledge, no existing research reported the prognosis and outcome of nocardiosis patients with infectious species numbers.

In this research, mNGS detected 9 out of the total 18 patients with mixed infections, outperforming the culture which only detected one species at a time. Previous studies have proved that mNGS is more sensitive than conventional methods in the detection of mixed infections, which was consistent with our study (Wang Jiahui and Feng, 2019; Fang Xiaowei et al., 2020; Chen et al., 2021). mNGS could detect mixed infections in one single test, prompting the clinical diagnosis and medication. We argue in favor of early mNGS in nocardiosis, to early identify the mixed infections and provide clinical warning, which may be a key step for improving patients’ outcomes.

It cannot be ignored that the sample size was small in our study (n=18). The small sample size might be attributed to the limited incidence of nocardiosis. Furthermore, as a new technology, mNGS has only recently been implemented in our hospital. We tried to incorporate all of the samples that met the inclusion criteria, and as a result, we included various sample types. Falsely negative error may occur due to the small sample (Faber and Fonseca, 2014). Rigorous statistical methods are needed in studies with smaller sample. Even so, small sample sizes may lead to more complex statistical problems in practical analyses. Therefore, our conclusions need to be verified in a larger sample size. Appropriate sample sizes should be estimated through sample size calculations to obtain accurate conclusions (Nayak, 2010). Another limitation was that as a retrospective study, antibiotic usage and patients’ clinical history might be not very accurate.

## Conclusion

In this study, we evaluated the clinical characteristics and infection status of nocardiosis patients who were positive for mNGS and clinically diagnosed as nocardiosis. We discovered that TATs of mNGS were faster than that of microbiological culture. Furthermore, mNGS outperformed culture in detecting mixed infections, and nocardiosis patients with mixed infections developed a higher risk of death. As a result, we believe that mNGS facilitates the early identification of pathogens, particularly mixed infections, which may be favorable to the prognosis of nocardiosis patients. Although the conclusion should be verified in the former study with an appropriate sample size.

## Data availability statement

The datasets presented in this study can be found in online repositories CNGB Sequence Archive (CNSA) of China National GeneBank DataBase (CNGBdb). The names of the repository/repositories and accession number(s) can be found below: https://db.cngb.org/search/project/CNP0002237/.

## Ethics statement

The studies involving human participants were reviewed and approved by the institutional review board of the First Affiliated Hospital of Zhengzhou University. The patients/participants provided their written informed consent to participate in this study.

## Author contributions

MJ, XM, and JL analyzed patients’ data and performed the experiments. MJ, YGL, HW, YL, and WG analyzed the genomics data. MJ and XM participated in the writing of the manuscript. JL designed the study and conceived the project. All authors read and approved the final manuscript.

## Funding

This work was supported by Chinese National Science and Technology Major Project [2018ZX10305410].

## Conflict of interest

The authors declare that the research was conducted in the absence of any commercial or financial relationships that could be construed as a potential conflict of interest.

## Publisher’s note

All claims expressed in this article are solely those of the authors and do not necessarily represent those of their affiliated organizations, or those of the publisher, the editors and the reviewers. Any product that may be evaluated in this article, or claim that may be made by its manufacturer, is not guaranteed or endorsed by the publisher.
