# Supplementary material for: Metagenomic next-generation sequencing provides prognostic warning by identifying mixed infections in nocardiosis
Source: Front Cell Infect Microbiol. 2022 Aug 31;12:894678. doi: 10.3389/fcimb.2022.894678 (PMC9471186; doi:10.3389/fcimb.2022.894678)
Supplement: Supplementary file 1 [file Table_1.docx]

Table S1 patients’ clinical information

| **case.No** | **Main symptoms** | ***Nocardia* infection sites** | **Underlying diseases** |
| --- | --- | --- | --- |
| 1 | fever, cough, chest tightness, chest pain, loss of appetite | pulmonary | hepatitis C |
| 2 | right abdominal pain, disorder of consciousness | disseminated (bacteremia) | hypertension |
| 3 | cough, fever, chest pain, chest tightness, expectoration | pulmonary | hepatitis C |
| 4 | cough, expectoration, fever, dyspnea, chest tightness, loss of appetite | pulmonary | chronic renal failure, coronary heart diseases, ANCA associated systemic vasculitis, bronchiectasis |
| 5 | cough, fever, hemoptysis, expectoration, chest tightness | pulmonary | hypertension, megaloblastic anemia, diabetes |
| 6 | fever, weakness | pulmonary | organizing pneumonia |
| 7 | fever, left shoulder and legs pain, expectoration, chest pain | disseminated (cutaneous, pulmonary) | nephrotic syndrome, hypertension |
| 8 | weakness, fever, hematuria | pulmonary | diabetes, hypertension, coronary heart diseases, myasthenia gravis |
| 9 | dyspnea, tachypnea | pulmonary | lung cancer with multiple metastases |
| 10 | swollen and pain of skin, weakness | disseminated (bacteremia, pulmonary) | nephrotic syndrome, diabetes |
| 11 | blood spots under the skin | disseminated (bacteremia) | diabetes, hypertension, immune thrombocytopenia |
| 12 | disorder of consciousness, fever | CNS | NA |
| 13 | fever, cough, expectoration, tachypnea | pulmonary | COPD, bronchiectasis |
| 14 | weakness, fever | pulmonary | systemic sclerosis, pulmonary fibrosis, polymyositis, coronary heart diseases |
| 15 | fever, chest tightness, weakness | pulmonary | diabetes, nephrotic syndrome |
| 16 | fever, expectoration, chest tightness | pulmonary | liver cirrhosis |
| 17 | fever, headache | CNS | NA |
| 18 | pus in the right ear, hearing loss | disseminated (bacteremia, pulmonary) | NA |

CNS, central nervous system; ANCA, antineutrophil cytoplasmic antibodies; COPD, chronic obstructive pulmonary disease.

Table S2 patients’ mNGS and culture results

| **case.No** | **sample type** | **mNGS results** | | | | **Culture results** |
| --- | --- | --- | --- | --- | --- | --- |
|  |  | **Nocardia species** | **Other bacteria** | **Fungi** | **Virus** |  |
| **1** | BALF | *Nocardia farcinica* | NA | NA | NA | *Nocardia* |
| **2** | Blood | *Nocardia asteroides* | NA | NA | NA | NA |
| **3** | BALF | *Nocardia cyriacigeorgica* | NA | NA | NA | NA |
| **4** | BALF | *Nocardia amamiensis  Nocardia pneumoniae* | *Enterococcus faecium  Staphylococcus haemolyticus  Streptococcus pneumoniae  Acinetobacter baumannii Eikenella corrodens* | *Candida tropicalis  Pneumocystis jirovecii Clavispora lusitaniae  Trichosporon asahii* | *Torque teno virus  Human betaherpesvirus 5* | *Candida tropicalis* |
| **5** | BALF | *Nocardia asiatica  Nocardia brevicatena  Nocardia brasiliensis  Nocardia abscessus  Nocardia exalbida  Nocardia niwae* | NA | NA | NA | NA |
| **6** | Lung tissue | *Nocardia cyriacigeorgica* | NA | NA | NA | NA |
| **7** | Abscess | *Nocardia farcinica  Nocardia cyriacigeorgica* | NA | NA | NA | NA |
| **8** | BALF | *Nocardia abscessus* | NA | NA | *Human betaherpesvirus 5* | *Nocardia* |
| **9** | BALF | *Nocardia abscessus* | *Enterococcus cecorum  Burkholderia cenocepacia* | NA | *Rhinovirus B* | NA |
| **10** | Blood | *Nocardia farcinica* | NA | *Pneumocystis jirovecii* | *Human betaherpesvirus 5  Human polyomavirus 1* | NA |
| **11** | Blood | *Nocardia brasiliensis* | NA | NA | NA | NA |
| **12** | CSF | *Nocardia carnea* | *Klebsiella pneumoniae* | NA | NA | *Klebsiella pneumoniae* |
| **13** | BALF | *Nocardia otitidiscaviarum* | NA | NA | NA | NA |
| **14** | BALF | *Nocardia cyriacigeorgica* | *Enterococcus faecium* | *Pneumocystis jirovecii* | *Human gammaherpesvirus 4   Human betaherpesvirus 5* | *Nocardia* |
| **15** | BALF | *Nocardia asiatica* | NA | *Pneumocystis jirovecii* | *Rhinovirus A   Human gammaherpesvirus 4* | NA |
| **16** | BALF | *Nocardia abscessus  Nocardia niwae* | NA | NA | NA | NA |
| **17** | CSF | *Nocardia carnea* | NA | NA | NA | NA |
| **18** | Blood | *Nocardia asteroides* | NA | NA | NA | NA |

CSF, cerebrospinal fluid; BALF, bronchoalveolar lavage fluid.

Table S3: antimicrobial drugs usage

| **case.No** | **Sulfonamides** | **Oxazolidinones** | **Carbapenems** | **Penicillin** | **β-lactamase inhibitors** | **Antifungal drug** | **Cephalosporin** | **Tetracyclines** | **Fluoroquinolones** | **Others** |
| --- | --- | --- | --- | --- | --- | --- | --- | --- | --- | --- |
| 1 | TMP-SMX | linezolid | biapenem, imipenem |  |  | voriconazole |  |  |  |  |
| 2 |  |  | biapenem, meropenem |  |  |  |  |  |  |  |
| 3 | TMP-SMX |  | biapenem | piperacillin | tazobactam |  |  |  | levofloxacin, moxifloxacin |  |
| 4 | TMP-SMX | linezolid | biapenem, imipenem |  |  | voriconazole |  | tigecycline |  |  |
| 5 | TMP-SMX |  | biapenem, imipenem, meropenem |  |  | voriconazole |  |  |  |  |
| 6 | TMP-SMX |  | imipenem | piperacillin | tazobactam |  |  |  | levofloxacin |  |
| 7 | TMP-SMX | linezolid |  |  |  | voriconazole | ceftriaxone |  |  |  |
| 8 | TMP-SMX |  | biapenem |  |  | voriconazole |  |  |  |  |
| 9 | TMP-SMX | linezolid | imipenem |  |  |  |  |  |  |  |
| 10 | TMP-SMX | linezolid |  | piperacillin | tazobactam |  |  |  |  |  |
| 11 | TMP-SMX |  |  |  | sulbactam | voriconazole | cefoperazone |  |  |  |
| 12 |  | linezolid |  |  |  |  | cefepime | tigecycline |  | rifamycins |
| 13 | TMP-SMX | linezolid | biapenem |  |  |  |  |  |  | rifamycins, clarithromycin |
| 14 | TMP-SMX |  | biapenem |  |  | voriconazole |  |  |  | clindamycin |
| 15 | TMP-SMX |  | biapenem |  |  |  |  |  | levofloxacin |  |
| 16 | TMP-SMX |  | imipenem | piperacillin | tazobactam, sulbactam | voriconazole | cefepime, cefoperazone | tigecycline | moxifloxacin |  |
| 17 |  |  |  |  |  |  | ceftriaxone |  |  | acyclovir |
| 18 | TMP-SMX |  | meropenem |  | avibactam |  | ceftriaxone, cefepime, ceftazidime | tigecycline |  |  |

TMP-SMX, trimethoprim-sulfamethoxazole.
